# Myeloid Sarcoma of the Colon Initially Presenting as a Paracolic Abscess in a Patient with Relapsed Acute Myeloid Leukemia

**DOI:** 10.3390/diagnostics14111062

**Published:** 2024-05-21

**Authors:** Seo Yeon Youn, Yu Ri Shin, Gyeongsin Park

**Affiliations:** 1 Department of Radiology, Seoul St. Mary’s Hospital, College of Medicine, The Catholic University of Korea, Seoul 14662, Republic of Korea; imseoyeon@gmail.com; 2 Department of Hospital Pathology, Seoul St. Mary’s Hospital, College of Medicine, The Catholic University of Korea, Seoul 14662, Republic of Korea; gspark@catholic.ac.kr

**Keywords:** myeloid sarcoma, acute myeloid leukemia, colon, abdominal abscess, hematologic neoplasms

## Abstract

Myeloid sarcoma, a rare extramedullary manifestation of acute myeloid leukemia (AML), can occur in various anatomic sites but seldom involves the gastrointestinal tract. We report the unusual case of a 49-year-old man with a history of AML who initially presented with abdominal pain and imaging findings suggestive of a paracolic abscess. However, the lesion rapidly progressed to a large descending colon mass with peritoneal involvement over five weeks. Surgical resection and histopathological examination confirmed a diagnosis of myeloid sarcoma. This case highlights the potential of myeloid sarcoma to mimic an inflammatory colonic process at initial presentation prior to manifesting as an overt mass lesion. Although exceedingly rare, myeloid sarcoma should be considered in patients with a history of AML presenting with colon lesions, particularly in those with an aggressive clinical course. Early recognition may expedite appropriate treatment and prevent unnecessary procedures. This report also underscores the importance of correlating imaging findings with clinical history and histopathology findings to establish an accurate diagnosis.

A 49-year-old man presented with intermittent, dull pain in the left lower quadrant of the abdomen for ten days. He visited our emergency room because of worsening symptoms. He had a history of acute myeloid leukemia (AML) with monosomy of chromosome 6, translocation between chromosomes 6 and 17, mono-allelic loss of TP53, and no commonly known molecular mutation 22 months previously. The initial diagnosis of AML was established through complete blood count (CBC) with differential, electrolytes, coagulation tests, flow cytometry immunophenotyping, and bone marrow histochemical staining. CBC revealed an elevated white blood cell count of 68.2 × 10^9^/L, predominantly composed of blasts, decreased hemoglobin (7.8 g/dL), and low platelet count (42 × 10^9^/L). Electrolyte levels and coagulation test results were within normal limits (INR 1.06). Flow immunophenotyping was positive for CD13, CD33, CD34, CD 64, and CD117, with partial HLA-DR expression. Bone marrow aspirate and biopsy confirmed hypercellular marrow with greater than 90% cellularity, predominantly composed of blasts. Histochemical staining was positive for myeloperoxidase and negative for Periodic acid-Schiff, confirming the myeloid lineage of leukemic cells. For the initial treatment of AML, the patient underwent induction chemotherapy with cytarabine (100 mg/m^2^, continuous infusion over seven days) and idarubicin (12 mg/m^2^ on days 1–3). After achieving complete remission, the patient received consolidation chemotherapy with high-dose cytarabine (3 g/m^2^ every 12 h on days 1, 3, and 5). The patient subsequently underwent allogeneic peripheral blood stem cell transplantation from an HLA-matched unrelated donor. No second induction chemotherapy was required prior to transplantation. Following allogeneic peripheral blood stem cell transplantation, the patient developed a chronic graft-versus-host disease affecting the skin, gut, and eyes, which was managed with immunosuppressive therapy. Physical examination revealed left lower abdominal tenderness. In addition to abdominal pain, the patient reported episodes of non-bloody diarrhea and occasional nausea, which did not respond to standard symptomatic gastrointestinal treatment. The patient did not report any constitutional symptoms, such as fever, night sweats, or unexplained weight loss, at the time of initial presentation or during the course of the disease. Laboratory studies showed mild elevation of C-reactive protein (5.35 mg/dL) and erythrocyte sedimentation rate (38 mm/h). Computed tomography (CT) revealed a 3 cm rim-enhancing fluid collection with adjacent inflammatory changes in the juxta-descending colon (Figure 1A). Colonoscopy yielded negative results, but a paracolic abscess was suspected based on the CT findings. After treatment with antibiotics, the pain resolved within 24 h. After five weeks, the patient returned due to progressively worsening abdominal pain. Physical examination revealed a hard, palpable mass in the left lower abdomen and diffuse abdominal tenderness. The patient was afebrile, with stable vital signs. Laboratory studies showed elevated C-reactive protein (11.95 mg/dL). Subsequent CT revealed an 8 cm enhancing soft tissue mass with perilesional fat infiltration involving the descending colon (Figure 1B). Additionally, a small amount of ascites with nodular peritoneal thickening was observed (Figure 1C). No bowel obstruction was caused by the large mass.

The patient underwent exploratory laparotomy, and a large mass was located in the descending colon, with severe adhesions to the peritoneum and adjacent small bowel loops. Multiple variable-sized nodules were found in the peritoneum and colonic mesentery. Conversion anterior resection of the descending colon mass and segmental small bowel resection due to adhesions and loop ileostomy were performed. The gross specimen showed a 12 cm ulceroinfiltrative yellowish mass (Figure 2A). There were small multifocal ulcers, but the majority of the overlying mucosa in this mass was intact. Histopathological examination revealed submucosal infiltration of immature myeloid precursors, with cytoplasmic granular positivity for myeloperoxidase (Figure 2B–D), consistent with myeloid sarcoma. Myeloid sarcoma showed a similar immunophenotypic profile and histochemical markers as the original AML. These findings support myeloid sarcoma as a relapse manifestation of AML.

After surgery, an 18F-fluorodeoxyglucose (FDG)-positron emission tomography (PET) scan showed intense FDG uptake along the peritoneum (Figure 3), which demonstrated extensive peritoneal sarcomatosis. Unfortunately, the patient’s condition did not improve significantly after extensive surgical procedures. Due to the poor condition, bone marrow biopsy and systemic chemotherapy, which are crucial for treating AML relapse and potentially colonic myeloid sarcoma, had to be postponed. He died of septic shock two months after surgery.

Myeloid sarcoma is a rare manifestation of immature myeloid cells at the extramedullary site [1]. It occurs more often in the skin, bone, and lymph nodes, although any part of the body may be affected. It may develop de novo or concurrently with hematological diseases, including AML. Myeloid sarcomas of the colon are extremely rare [2]. The CT imaging features of myeloid sarcomas involving the gut are variable and nonspecific [3]. Myeloid sarcomas of the gastrointestinal tract may be asymptomatic or present with abdominal pain, small bowel obstruction, diarrhea, or bleeding [4]. It may be impossible to initially diagnose myeloid sarcoma, which can lead to diagnostic challenges. Because it is a rare disease, a consensus on myeloid sarcoma treatment has not been achieved. However, the most commonly recommended treatment for myeloid sarcoma is the conventional AML protocol [5]. Excision or debulking surgery may be considered before initiating chemotherapy. Another role of surgery is to obtain pathological confirmation in cases that are difficult to diagnose. However, an aggressive surgical approach with multivisceral resection has not been supported. In this case, if myeloid sarcoma is suspected by a colorectal surgeon, only diagnostic laparotomy should be performed, not an aggressive surgical approach for anterior resection.

To the best of our knowledge, no case of myeloid sarcoma of the colon has been reported to be a rapidly growing paracolic inflammatory lesion. This is the first report of myeloid sarcoma in the colon with an unusual presentation mimicking a paracolic abscess as the initial manifestation of relapse of acute leukemia. Although no imaging pattern is pathognomonic for colonic myeloid sarcoma, understanding the imaging appearances of myeloid sarcoma manifestations can help radiologists establish diagnostic considerations, especially in patients with a history of hematological disorders and complaining of a rapidly growing palpable mass in the colon.

## Figures and Tables

**Figure 1:**
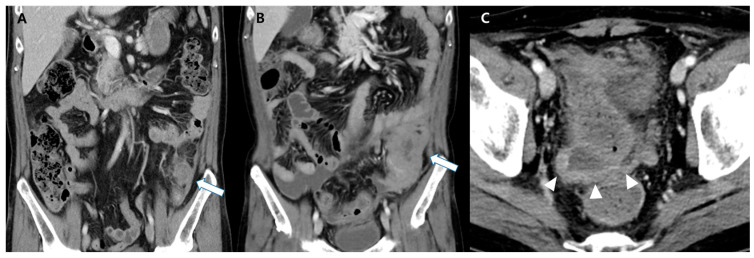
(**A**) Initial contrast-enhanced computed tomography image. This image shows rim-enhancing fluid collection with adjacent inflammatory changes localized in the juxta-descending colon, mimicking a paracolic abscess (arrow). (**B**) Follow-up scans obtained five weeks later. This follow-up image demonstrates the rapid and unexpected development of a large soft-tissue mass (arrow) in the descending colon (previously the site of the suspected abscess). Additionally, a small amount of ascites with nodular peritoneal thickening was observed (arrowheads). This finding prompted further investigations. (**C**) Additionally, a small amount of ascites with nodular peritoneal thickening was observed (arrowheads). This finding prompted further investigations.

**Figure 2:**
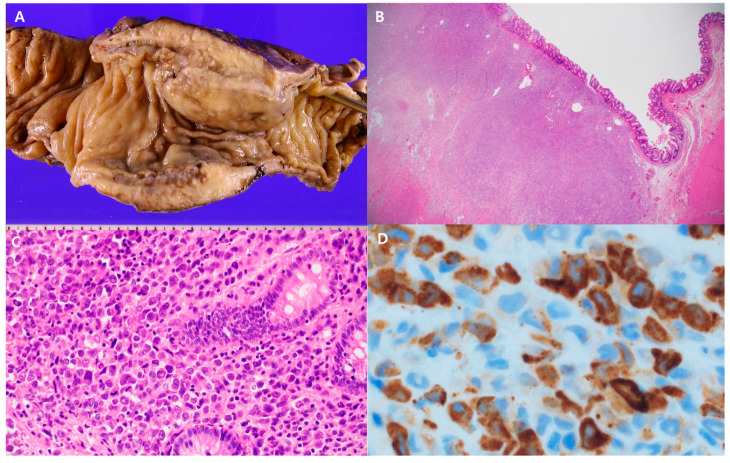
(**A**) Gross image of the resected colonic segment. This specimen revealed an ulceroinfiltrative 12 cm yellowish mass encircling the descending colon (**B**,**C**). Histological examination revealed submucosal tumor infiltrates and normal colonic glands. These panels show microscopic findings at different magnifications (20× and 400×) stained with hematoxylin-eosin. The images revealed submucosal infiltration by immature myeloid cells with a normal overlying colonic mucosa. (**D**) Immunohistochemistry with myeloperoxidase staining. The neoplastic cells exhibited cytoplasmic granular positivity on myeloperoxidase immunohistochemical staining, confirming myeloid differentiation (400×).

**Figure 3:**
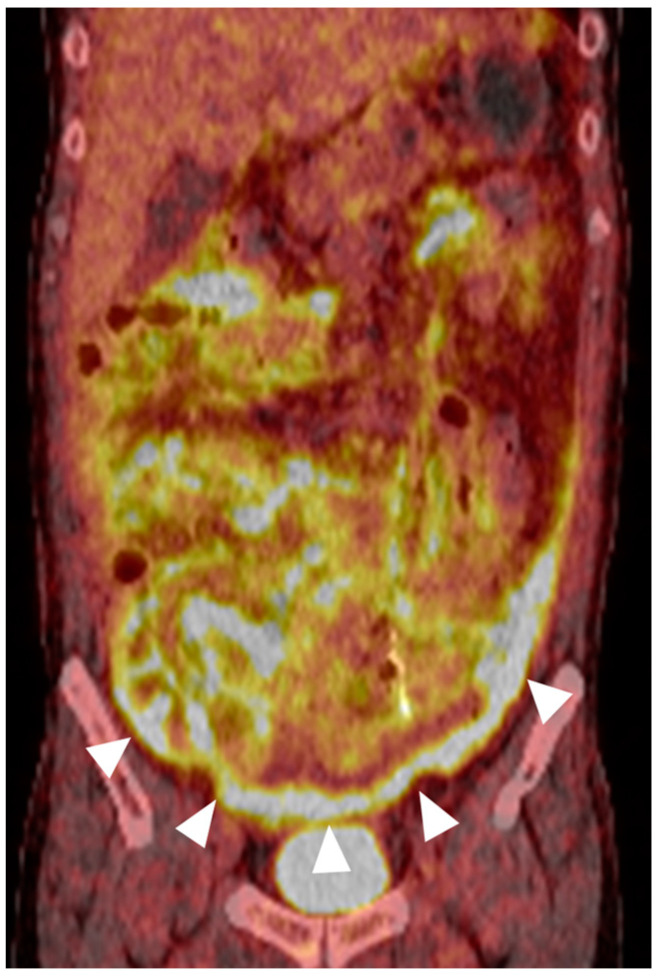
^18^F-FDG-PET/CT imaging. This coronal image from an ^18^F-fluorodeoxyglucose (FDG)-positron emission tomography (PET) scan reveals diffuse FDG activity along the majority of the peritoneal lining (arrowheads) to a degree much higher than the physiological hepatic uptake. This finding was consistent with widespread peritoneal sarcomatosis.

## Data Availability

Not applicable.
